# Smart wearable body sensors for patient self-assessment and monitoring

**DOI:** 10.1186/2049-3258-72-28

**Published:** 2014-08-22

**Authors:** Geoff Appelboom, Elvis Camacho, Mickey E Abraham, Samuel S Bruce, Emmanuel LP Dumont, Brad E Zacharia, Randy D’Amico, Justin Slomian, Jean Yves Reginster, Olivier Bruyère, E Sander Connolly

**Affiliations:** 1 Neurodigital Initiative, Columbia University, Department of Neurological Surgery, 630 West 168th Street, New York, NY 10032, USA; 2 The Joan and Irwin Jacobs Technion-Cornell Innovation Institute, Cornell NYC Tech 111 8th Avenue #302, New York, NY 10011, USA; 3 Bartoli Brain Tumor Research Laboratory, Columbia University Irving Cancer Research Center Columbia University Medical Center, 1130 St. Nicholas Ave., New York, NY 10032, USA; 4 Department of Public Health, Epidemiology and Health Economics, University of Liège, Liège, Belgium and Support Unit in Epidemiology and Biostatistics, Department of Public Health Sciences, University of Liège, Liège, Belgium

**Keywords:** Sensors, Mobile health, eHealth, Patient education, Quantified patient

## Abstract

**Background:**

Innovations in mobile and electronic healthcare are revolutionizing the involvement of both doctors and patients in the modern healthcare system by extending the capabilities of physiological monitoring devices. Despite significant progress within the monitoring device industry, the widespread integration of this technology into medical practice remains limited. The purpose of this review is to summarize the developments and clinical utility of smart wearable body sensors.

**Methods:**

We reviewed the literature for connected device, sensor, trackers, telemonitoring, wireless technology and real time home tracking devices and their application for clinicians.

**Results:**

Smart wearable sensors are effective and reliable for preventative methods in many different facets of medicine such as, cardiopulmonary, vascular, endocrine, neurological function and rehabilitation medicine. These sensors have also been shown to be accurate and useful for perioperative monitoring and rehabilitation medicine.

**Conclusion:**

Although these devices have been shown to be accurate and have clinical utility, they continue to be underutilized in the healthcare industry. Incorporating smart wearable sensors into routine care of patients could augment physician-patient relationships, increase the autonomy and involvement of patients in regards to their healthcare and will provide for novel remote monitoring techniques which will revolutionize healthcare management and spending.

## Background

Innovations in mobile and electronic healthcare are revolutionizing the involvement of both doctors and patients in the modern healthcare system by extending the capabilities of physiological monitoring devices [1,2]. Expansion of health information technology and consumer e-health tools and services, such as telemonitoring platform and mobile health applications [3], have created new opportunities for individuals to participate actively in their healthcare, and provides the opportunity for remote monitoring of clinically relevant variables in non-clinical settings [4]. These devices can be integrated into routine care of acute and chronic diseases and provides essential information for management to both the healthcare providers and patients [5]. Studies show that a well-informed patient improves quality of life and patient outcome because they are more likely to participate in healthy behavioral changes [6,7]. Furthermore, the United States spends approximately 75% of their $2 trillion budget on chronic diseases per year, which make up 7 out of 10 deaths annually [8]. Chronic diseases also have debilitating effects, which lead the nation in causes of major disabilities and preventable illnesses [8].

The concept of remotely monitoring patients is not new but recently a lot of attention has been placed on smart wearable body sensors (SWS) [4,9]. Whereas other articles have focused primarily on devices which have been used for research or have needed a physician’s prescription, this article expands upon the opportunities and studies with devices that are available to all consumers. There is now more evidence to support the reliability of these devices and the technology is more easily accessed. These devices contain an assortment of different sensors which can be used to monitor variables and transmit data either to a personal device or to an online storage site. The variety of the sensors can be attributed to the types of stimuli that they respond to (e.g. physiological vital signs, body movements, and organic substances) and their placements (clothing, subcutaneous implant, body part accessory, etc.) These devices have the opportunity to meet the patients’ needs by administering information in real-time to the patient’s smartphone, computer or other wireless devices and has the potential to influence their behaviors [5,6]. Sensors allow patients to self-monitor, track, and assess human physiological data, while also providing interfaces and a dashboard for healthcare providers [7]. These sensors are easily managed and are becoming increasingly accurate and reliable for patient care [5,10,11]. The SWS’s can also be utilized as a diagnostic tool to aid in identifying and managing a myriad of diseases [7]. Current sensor technology for vital-sign monitoring promises great benefits for prevention, prediction, and management of diseases. Despite significant progress within the monitoring device industry, the widespread integration of this technology into medical practice remains limited.

The purpose of this manuscript was to evaluate existing wearable sensors and describe their current medical applications.

We therefore used general search engines such as Pubmed, Science Direct, and Google Scholar to extensively search for “wearable sensor”, “mHealth”, “eHealth”, “medical sensor”, “Personal Area Network”, “Body Area Network”, “Body Sensor Networks”, “Tracker”, “Monitoring”, “Self Tracking” and combination of these terms. The search was performed using pertinent Medical Subject Heading terms. We reviewed these studies in order to present clinical utility.

### Wearable body sensors

The SWS include a wide range of wearable devices and sensors such as accelerometers and gyroscopes, smart fabrics and actuators, wireless communication networks and power supplies, and data capture technology for processing and decision support [12]. Having a wearable device decreases the restrictions placed on their motility and daily activities which allows monitoring in the environment of the patients directly home but also at work.

The most used and well-known sensor accelerometers are electrochemical sensors that measure acceleration of objects in motion along reference axes and provide basic step and activity counts used as a quantitative assessment of physical activity [11,13]. This data can be used to obtain velocity and displacement by merging the data with respect to time [5]. Triaxial accelerometers, which monitor vibrations in three planes, can detect movement and posture, such as upright or lying down, according to the magnitude of acceleration signals along sensitive axes [14,15]. Gyroscopes are also another popular type of sensor. A gyroscope is a mechanical device that measures 3-D orientation based on the principles of angular momentum. A spinning rotor tends to maintain its orientation allowing the changes in orientation to be calculated by integrating the angular velocity [14].

Placement of SWS is versatile and provides flexibility and comfort for patients, which is one of the keys for patient acceptance. There are many devices already on the market for fitness and wellness that use consumer-facing applications which can be easily incorporated into clinical practice. Most sensors can either be worn or placed on clothes. Some wearable devices can be placed on the almost any part of the body: wrist, ankle, waist, chest, arm, legs, etc. These sensors can detect many different variables such as speed, distance, steps taken, floors climbed and calories burned [16]. Implementation of a real-time waist-mounted tri-axial accelerometer unit detects a range of basic daily activities, including walking and posture [17,18]. Other possibilities for wearable sensor placement include gloves, rings, necklace, brooches, pins, earrings, and even belt buckles. These models have been used to monitor blood oxygen saturation (SpO2), heart rates, and record hand posture while manipulating objects, such as eating or dressing [19,20]. A newly marketed device measures body temperature through the use of an ear probe which detects infrared radiation from the tympanic membrane [21]. Another approach which could be more convenient for patients is the placement of sensors in clothing, such as a vest or shoe. Smart Vest is a wearable physiological monitoring system for parameters such as, heart rate, blood pressure (BP), body temperature, galvanic skin responses, and can even perform electrocardiograms (ECG) [22]. There are also experimental designs, with promising preliminary results, demonstrating that sensors (heart rate, acceleration, and respiratory activity) can be incorporated into a regular t-shirt rather than a bulky vest, which adds another layer of convenience [23]. Placement in the shoe can provide a more convenient method to measure differences between mean foot extreme and gait stride time for healthy gait and those with physical disorders, as well as proved highly capable of detecting foot orientation and position [22].

### Self-tracking and monitoring

Traditionally there have been three widely accepted approaches for outpatient monitoring: patient reported outcomes (PRO), telemonitoring and quantifying self-hybrid models (QSHM) [24]. PRO models encourage the patient to be proactive by allowing them to have more autonomy. This is accomplished by having the patient self-report a descriptive analysis of subjective data to their provider. Unfortunately, this data can be unreliable and inconsistent for objective measurements. Telemonitoring uses equipment to monitor physiological data passively, which is then transmitted to the patient and can also be sent to their provider. This monitoring could extend or replace routine outpatient care in dedicated hospital wards. Whereas PRO models report subjective data, telemonitoring can be used to report objective data but a limitation to this technology is that it is only capable of reporting quantifiable variables. Lastly, QSHMs have been developed in order integrate the previous methods by allowing the patient to be able to report their non-quantifiable variables while still having the ability to monitor quantifiable ones. This amalgamation mitigates the data that may be unreliable from the self-reporting coming from the patients and bypass the limitations of variables in the telemonitoring model. This model provides the ability for a patient to better understand their healthcare by integrating complement models that combine subjective symptoms with objective criteria. The majority of SWS fall under the telemonitoring model but a few possess the ability to allow the user to input subjective data as well, which then follows the QSHM model. Ideally, SWS would continue this trend towards QSHMs.

Many individuals with chronic diseases could benefit from having constant remote monitoring and the best way to monitor a patient is through understanding their interactions with their daily activities. Giving the patient the opportunity to depart from the hospital and continue to monitor themselves will allow for a more authentic representation and a more accurate assessment of physiological data. If patients could be monitored reliably away from the hospital, this could decrease the cost associated with the length of stay (LOS), which can greatly decrease healthcare costs and unintended consequences. Shifting the paradigm from a culture of treatment to one centered upon prevention.

### Overview of direct clinical applications

#### Cardiopulmonary and vascular monitoring

The increase in reliable monitoring and reporting coupled with the versatility of sensor placement has facilitated efforts to implement SWS in clinical settings. Most of the attention to date has been focused on blood pressure monitoring with at home using sensors. The European guidelines on cardiovascular disease (CVD) prevention recommend frequent blood pressure monitoring in order to prevent coronary diseases [25]. The dominance of chronic diseases as the major global death contributors has emerged, and The World Health Organization (WHO) estimates there will be about 20 million CVD deaths in 2015, accounting for about 30 percent of worldwide deaths [26,27]. Additionally, telehome monitoring has been demonstrated to improve quality of care in patients with CVD [28]. These statistics stimulated great demand worldwide for a device that detects respiration rate, breathing patterns and fatal breathing changes for prevention of or early detection of CVD [28]. A new non-invasive long-term blood pressure measurement device measures the BP continuously on the wrist using ultrasound, a small balloon, and an actuator [29]. A ring sensor has also been used to facilitate the management of hypertension and congestive heart failure [12].

Continuous multimodal measurement devices have also been developed. The Advanced Medical Monitor (AMON) system is a wristwatch model with a multi-variable sensor device [30]. AMON contains an accelerometer that continuously measures physical activity and comprises other sensors in order to monitor BP, blood oxygen saturation, body temperature, and can take an ECG [31-33]. Another wrist module was developed to measure BP by integrating a photoplethysmographic (PPG) sensor and an ECG sensor, allowing for continuous monitoring [34,35]. The Murata vital sign sensor uses the optical absorption of hemoglobin proteins to make measurements of pulse and blood oxygen levels, and has two electrodes that measure the voltage differences generated by the heart. Murata’s innovative algorithm also has the ability to estimate user fatigue levels and exercise stress [36].

Multiple types of cardiac monitoring devices exist. Some involve surgical implantation of wireless devices that can monitor and report data to a smart phone and other devices can give patients access to a 24 h ECG via an adapter that acts as a phone cover. Most of the devices are external and can be placed on the wrist or around the thorax to accurately monitor cardiac function. An example of an external device is AliveCor’s integrated phone case and ECG leads, which allows patients to monitor and record their cardiac rhythms, as well as send their information to healthcare providers [37]. This device has been cleared by Food and Drug Administration (FDA) as a Class II device and has received approval of their 510(k) in order to be available on the market. The University of Southern California recently published an article regarding its reliability and utility of this device within the cardiovascular field [38]. With increasing computational capacity, storage capacity and ubiquitous connectivity, smart phones enable individuals to actively monitor their health in new locations [37]. Doctors could use these types of devices in order to diagnose early signs or cardiac abnormalities that could potentially lead to better outcomes for cardiac patients. Atrial fibrillation (Afib) is the most common cardiac disorder and may be asymptomatic. Most patients are not diagnosed with Afib until their condition worsens to the point of heart attack, angina, stroke or heart failure. A recent study compared AliveCor’s device to a standard 12-Lead ECG to see if it was suitable for screening silent Afib, and found that the sensitivity and specificity were high and provided accurate and reliable data [39]. The cornerstone of Afib management is simply early detection and SWSs facilitate this.

Studies at Mayo Clinic using “off-the-shelf” monitors have demonstrated the reliability and utility of accelerometers to assess mobility in the elderly after surgery [10]. In this study, they used wireless accelerometers on postoperative cardiac surgery patients and found a correlation between the number of steps taken in their early recovery period, length of stay, and dismissal disposition [10].

Other multimodal sensors can monitor respiratory rate and concurrently monitor oxygen saturation, coughing events, and other respiratory variables. A good example of this type of monitoring is the microwave reflectometric vital signal sensing systems which have been developed to detect very weak microwaves that irradiate and scatter off the human body [40]. A sensitive microwave sensor monitors the reflected waves, which change in phase in response to motions of the body, including the regular displacement of the chest during breathing or, the slight movement of the chest caused by the beating heart. This illustrates that these devices have an integrated system of sensors that can be used to monitor different variables rather than needing separate devices for each individual variable. A recent measurement system for drivers was introduced, where measuring the pressure applied to a gauge embedded in a seat belt derives the respiration rate [41]. Universal Biosensors of Melbourne Victoria have also been working on a creating an electrochemical sensor that can measure prothrombin time for those patients who are taking warfarin [42].

#### Glucose home monitoring

While many self-management phone applications have been flexibly tailored to individual health requirements through a routinely carried item, much research has been invested in determining blood glucose levels with wearable sensors [43]. A non-invasive continuous self-monitoring device could greatly increase the patient’s autonomy and improve the efficacy in the management of diabetes [44]. Arm modules developed by Solianis Monitoring AG are a multi-sensor approach. The medium-length and long electrodes penetrate to deeper layers of tissue, providing information related to changes in glucose levels; the short electrode penetrates to the surface layer, providing data related to other parameters such as temperature and humidity [45].

A recent clinical trial sponsored by the University of Virginia studied the feasibility of a portable pancreas system in patients with type one diabetes mellitus [46]. Their method utilized a combination of a monitoring device and insulin pump to monitor their patients, along with a computer algorithm that incorporated a closed-loop control platform via a smart phone to modulate the concentration of insulin. Twenty participants were monitored over a 42 hour period and this study demonstrated that a smart phone was capable of operating as an outpatient closed-loop control device, which was comparable to an inpatient setting using a laptop configuration. This study supports the idea that physicians can accurately monitor their patients remotely and increases the patient’s quality of life by allowing a method of constant monitoring without having to check their glucose levels periodically.

A recent innovation, that further accommodates individuals that are constantly monitoring their glucose levels, is a non-invasive ocular glucose sensor. Although this technology is still under development, many companies are creating contact lenses that have an integrated diagnostic sensor that detects glucose levels and transmits them to a personal device. Some versions of this glucose monitor use tears as a source of energy [47]. Google has recently taken their prototype to the FDA for early independent clinical trials [48]. Their product checks the ocular glucose levels every second and they are also experimenting with placement of a light-emitting diode (LED) light that will shine when glucose levels are not within a particular range. Although this technology is still years away from being released for consumers, it acknowledges that there is a demand from the consumer for more convenient methods to increase their quality of life.

#### Neurological function monitoring

One area that has great potential and has had success with SWS is neurological monitoring; specifically in post-operative management, outpatient care and rehabilitation medicine. These sensors have the ability to seamlessly analyze gait, limb paralysis, cerebral palsy, and have diagnostic capabilities such as, early detection of Parkinson’s (PD) and Alzheimer’s disease (AD). A study in 2009 used two different sensors, AMP 331 and Minimod, one placed on the lower back and another placed superior to the right ankle, to monitor gait in children with cerebral palsy (CP) and compared them to matched controls [32]. This study determined that sensors are a reliable method to monitor gait in children with CP and also demonstrated that not all sensors are accurate and reliable. The Minimod sensors were more reliable and accurate when monitoring the average stride length and step count with both groups compared to the AMP sensors, suggesting that more research needs to be conducted to ensure that the correct information is being used. Another study conducted in 2011 using inertial sensors that provided auditory and visual feedback demonstrated a rehabilitative approach to sensors [49]. This study demonstrated that patients with CP related gait disorders had a 21% residual short-term improvement in walking speed and 8% increase in stride length with visual feedback, as well as an average short-term improvement of 25% in walking speed and 13% in stride length for auditory feedback. The results were compared to matched controls, which did not show a measurable change in gait [49].

Sensors can also be used to monitor seizure activity in patients. Preliminary studies conducted at Stanford University Medical Center demonstrated that a wristwatch style device named “SmartWatch" was able to detect seven out of eight total seizures and accurately transmitted this information to the patient’s caregiver [50]. While “SmartWatch” cannot predict seizures, this sensor allows caregivers to be alerted and react more quickly, decreasing the probability of serious injury or death. Accelerometers have also been used as a reliable and objective device to monitor the free-living physical activity of 40 stroke patients [51]. Rand et al., monitored subjects for three consecutive days and on a 6-minute walk and found these devices to be consistent. They also mention that the physical activity was very low, with 58% of the participants not meeting the recommended activity levels. This pertinent information provides both the provider and patient the objective data needed in order to modulate and manage treatment in an effective manner while allowing the patient the ability to stay home.

Sensors have also played a critical role in the detection of Alzheimer’s Disease. Pathologically, these patients undergo degeneration of the suprachiasmatic nucleus, which effects circadian pace makers, which lead to an impairment of temporal structure with motion behavior. Researchers from Computer Science and Electrical Engineering of Rostock University and the German Center for Neurodegenerative Diseases (DZNE) Rostock, placed three-axis accelerometers on the ankles of 23 dyads (n = 46) consisting of diagnosed Alzheimer’s patients and healthy control subjects [52]. The subjects were chosen from a sample of community dwelling individuals and were matched according to age, gender and education. A trained medical student placed the sensors on the dyads on the first day and would then remove them on the third day, allowing for continuous monitoring of the subjects. The novel algorithm was able to discern unlabeled Alzheimer’s patients from healthy control subjects 91% of the time; this coincides with a higher rate than the conventional Cohen-Mansfield Agitation Inventory [52]. The author’s state that the higher accuracy denoted in this study suggests that the spectral structure is associated with clinical diagnosis of AD.

Much research has also been invested in monitoring patients with PD. The contemporary clinical assessment does not adequately reflect the patients’ actual status during daily life. Weiss et al. initiated a project to assess mobility in patients with PD, with the use of SWS [53]. Healthy adults and patients with PD wore a triaxial accelerometer on their waist during short walks. They used frequency-domain measures to quantify gait variability in the daily living environment. The average stride time was statistically significant for the PD patients than for the controls and the walking patterns of the PD patients were less consistent. Average stride times were reported as being highly correlated to the dominant frequency. Weiss concluded that frequency-based measures and sensitive estimates to stride-to-stride variability could serve as an objective, easily calculated marker of gait variability in real-world settings and the ease of the sensor placement and monitoring allows this to be a feasible option.

Neurological function monitoring has been successful in these applications and can be extended towards other populations of diseases and disorders. The aforementioned studies demonstrate the clinical relevance and the potential for clinical utility. The integration of SWS allows the physicians to be able to detect earlier diseases, monitor and modulate recovery of patients and create novel therapies for rehabilitation.

#### Physical therapy and rehabilitation

The SWSs are able to monitor mobility in specific therapeutic exercises designed for rehabilitation to provide objective criteria of the progression of the patient. This information can be used to help improve exercise techniques, thereby aiding the patient to maximize therapeutic recovery. The wearable health-monitoring device can be integrated into a user's clothing and performs real-time analysis of sensors' data, provides guidance and feedback to the user, and can generate warnings based on the user's state, level of activity, and environmental conditions [54].

Sensors have also been integrated into pulmonary rehabilitation. This includes graded exercises, strength and flexibility training, collaborative self-management education, and has been shown to improve physical functioning and life quality. Sensors are now considered an integral component of optimal care for people with severe lung disease [52]. Steele et al., who provide an overview of the potential utility of motion sensors to measure physical activity in people with chronic pulmonary diseases, concluded that SWS, specifically accelerometers, have considerable potential for answering questions related to pulmonary rehabilitation, such as how to encourage participants to engage in exercise and increase their overall activity [55]. Accelerometers can measure the efficacy adherence intervention of patients following a pulmonary rehabilitation program to promote physical activity and exercise following program completion. While most of the research has been devoted to sensors involving therapy, sensors for prevention and early detection as diagnostic tools are becoming increasingly active.

#### Prevention and motivational aspects of sensors

The numerous advancements in SWS are primarily due to the “quantified self” [4]. The quantified self is a movement to incorporate technology regarding healthcare into regular data acquisition to create more transparency and customization, give the patients greater access and decision making regarding their health, and improve healthcare systems overall. The quantified self is evolving the role of the patient from a minimally informed recipient, to an active collaborator by creating better doctor-patient partnership models [4]. The main focus of the evolving health care model has, thus far, been in regards to therapeutic and treatment models for sick patients. However, more patient driven health resources, like SWS, are converging to produce a trend of increased personal health surveillance and monitoring so healthy consumers can become empowered to make healthy choices as preventative measures. According to Pew Internet Research, who carried out the first U.S. national self-tracking survey, 69% of U.S. adults track at least one health indicator for themselves, or a loved one and approximately half of them stated that tracking these variables has changed their overall approach to health [39]. Out of the 3014 subjects surveyed, 60% stated they tracked their weight, diet, or exercise routines, 33% tracked health indicators or symptoms like blood pressure, blood sugar, headache, or sleep patterns, and 12% tracked health indicators or symptoms for a loved one. This demonstrates a growing interest from the population to being able to access this information. The SWS and other emerging patient driven technology are particularly focusing on the earlier stages of healthcare, targeting prevention rather than reacting towards unfavorable outcomes. Consumer reflection on SWS data could be extended to innovative perspectives to the overall consideration of health care. The patient can become more of an informed participant and take active responsibility in their health by taking healthy preventative measures. By providing health management data, the quantified self engages healthy patients in a variety of self-tracking and management methods that can be utilized for disease prevention, further developing the overall health care system.

## Discussion

Table 1 summarizes the sensors that were reviewed in this article. This narrative review intended to provide an overview of the clinically relevant developments and utility of SWS. While increasing access to monitoring devices for patients has great potential to augment healthcare, this information can be misunderstood and misused.

**Table 1:** Review of sensors

**Clinical applications**	**Location**	**Type of Sensor**	**Marker**
Cardiopulmonary & Vascular Monitoring	Wrist	Ultrasound [29]	Blood pressure
		Multi-variable (AMON) [33]	Blood pressure, blood oxygen saturation, body temp., heart rhythm
		Photoplethysmographic and electrocardiograph [34,35]	Blood pressure and heart rhythm
	Finger (ring sensor)	Optical (heart rate)	Heart rate and temperature
		Radio-frequency identification (pulse & temp.) [12]	
	Arm or thigh	Microwave reflectometric cardiopulmonary [40]	Heart rate variability as a method to evaluate stress
	Can be used with various equipment units	Optical absorption sensor	Blood oxygen, heart rate and rhythm, fatigue levels and exercise stress
		ECG electrodes [36]	
	Phone adapter	Single-channel ECG [37]	Heart rate and rhythms
	Seat belt of a car	Wire-type strain gauge [41]	Heart rate and respiration rate
Glucose Home Monitoring	Arm	Multi-variable [45]	Blood glucose
	Subcutaneous	Glucose [46]	Tissue glucose
	Eye	Glucose [48]	Ocular glucose
Neurological Function Monitoring	Clothes	Inertial sensors and accelerometers [32,51-53]	Walking distance, stride distance, and step count during stair ascent and descent
	Visual feedback-glasses	Inertia [49]	Walking speed and stride length
	Auditory feedback-headphones		
	Wrist or ankle	Accelerometer and motion [50]	Seizure activity
Physical Therapy and Rehabilitation	Ankle	Pedometers and accelerometer [10,55]	Walking distance and step counts

A major barrier for the implementation of SWS is the reliability and efficiency of sensor systems and data processing software [56]. Some of the studies reported in this review had authors who were also the system developers. This could lead to positive biases for their products and the dearth of randomized clinical trials, either for practical reasons or logistical ones, makes it difficult to truly scrutinize the results. However, many studies are being conducted to improve the reliability of sensors [56,57]. Smart wearable sensors, specifically accelerometer-based devices, have undergone many trials to determine their accuracy and precision. While accelerometers in a broad sense have been proven effective [58], individual studies and devices each require mean and variance determinations and adjustments to gain the most accurate results for the desired values. Some studies have even used multiple sensors on patients to combine data to achieve optimal results. A study by Olguin and Pentland compared the activity recognition accuracy of four configurations of accelerometers from three placements; the chest, wrist, and hip [59]. The mean and variance of the three axes were used as inputs to a Hidden Markov model. The classifier achieved an accuracy of 65% using only one accelerometer placed at the chest. By combining data from accelerometers placed on the wrist and hip, the accuracy increased to 87%. They also found that it is possible to obtain similar results using only two accelerometers placed on the chest and hip. Other chest-worn accelerometers are able to detect respiratory and snoring features for sleep apnea diagnosis [60].

Many SWS employ algorithms to transform data obtained and many times the results are only estimates of the physiological data. There are a myriad of variables that could influence the estimates and their generalizability needs to be confirmed by the physicians and patients. This review attempted to use articles that demonstrated real-world applications of these devices rather than studies which have only been used in laboratories. The optimal application of these devices would be to tailor each one to the individual using them and regularly calibrate whenever necessary. Although healthcare is always trying to increase patient’s autonomy and create a harmonious relationship between physicians and patients, endowed with this technology some patients could erroneously disregard the role of the physician. This could be circumvented through patient education and understanding of the limits of this technology. There is no “one size fits all solution”, and matching the right technology for a given patient population or desired clinical objective is key to ensuring sufficient perceived usefulness and uptake [61-63]. Furthermore, with sensors that operate on closed-loop systems, such as the aforementioned sensors for diabetic patients and AD, the adaptability of these SWS can be used as a method to provide personalized medicine to patients in novel ways that were not available before. Rather than strict monitoring, these devices have the ability to calculate idiosyncratic patterns that can be used to modulate treatment and tailor it to the specific needs of the individual. As access to these devices continues to increase, the feasibility of more direct comparisons of these devices will be available.

Additionally, SWSs can still be very expensive and, to the best of these authors’ knowledge, there have yet to be a designation of codes for reimbursement of these devices [64]. We believe that as the trend to utilize these devices by patients and physicians continues to rise, eventually these will be integrated into the coding systems that will allow for reimbursement of products for consumers. With the increase in physician shortages, some states, such as Maryland, have already started to expand coverage through telemedicine for delivery of health care services [65].

Legal and ethical issues such as privacy data protection and ownership are also major concerns of any Internet-based application. The balance between the patient as the owner of data and the documentation and use of the data must be properly managed, with patient confidentiality always at the forefront without impeding the development of innovative solutions. Moreover, researchers believe that SWSs introduce risks of social inclusion of users [66]. Lastly, elderly users strive for independence and any technology that seems to limit their independence will be met with opposition [67].

## Conclusion

The evolution of SWS and their ability to track mobility, health indicators, and symptoms have great potential that can revolutionize the healthcare system and change patient behavior. Driven by the quantified self, emerging patient driven healthcare models are contributing to shaping a positive future for healthcare with the patient at the epicenter. Rather than a physician reacting to an event that occurred to a patient, the SWS distributes responsibility to the patients which can lead to more personalized medicine. There has already been a host of clinical applications involving SWS that have been analyzed, including but not limited to blood pressure, cardiac monitoring, respiratory rate, blood electrolyte and glucose concentration systems, neurological monitoring, and physical therapy and rehabilitation medicine. These technologies are continuously being improved upon and can extend into any field of medicine. However, the integration of wireless technologies requires an infrastructure of evidence regarding reliability, validity, and responsiveness for each application across a range of disease and injury related disorders while also contributing to preventative methods. Collaboration between physicians, patients, engineers, and the wireless industry is essential for the design and optimization of inexpensive wireless systems. Further studies and clinical trials are needed to further this research and provide better overall models for patients. The quantified self is being pioneered by the patients and is revolutionizing patient behavior as they adopt healthy behavioral changes into preventative measures. These changes will alter the way that countries utilize funds on healthcare, set guidelines for protocols regarding preventative and post-operative monitoring, and augment the physician-patient relationship. Incorporating these technologies now will facilitate the transition and increase favorable outcomes in the future.

## Competing interests

The authors do not have any potential or actual competing interest.

## Authors’ contributions

All authors have contributed significantly to this manuscript. All authors have contributed to either (1) the conception, design, and acquisition of data (GA, EC, MA, SB, ED) (2) drafting the article (GA, MA, EC, SB, ED, BZ, RD, JS, OB, JYR, ESC) and (3) revising it critically for important intellectual content (GA,OB, JYR, ESC). All authors read and approved the final manuscript.
